# Running towards injury? A prospective investigation of factors associated with running injuries

**DOI:** 10.1371/journal.pone.0288814

**Published:** 2023-08-17

**Authors:** Sarah Dillon, Aoife Burke, Enda F. Whyte, Siobhán O’Connor, Shane Gore, Kieran A. Moran

**Affiliations:** 1 School of Allied Health, University of Limerick, Limerick, Ireland; 2 School of Health and Human Performance, Dublin City University, Dublin, Ireland; 3 Insight SFI Research Centre for Data Analytics, Dublin City University, Dublin, Ireland; 4 Centre for Injury Prevention and Performance, Athletic Therapy and Training, Dublin City University, Dublin, Ireland; The Wingate College of Physical Education and Sports Sciences at the Wingate Institute, IL, ISRAEL

## Abstract

**Background:**

Given the high incidence and heavy burden of running related injuries, large-scale, prospective multifactorial investigations examining potential risk factors are warranted. This study aimed to identify factors associated with running related injuries and to evaluate their potential in injury screening.

**Study design:**

Prospective cohort study.

**Materials and methods:**

Two hundred and seventy-four recreational runners were recruited. Clinical measures (strength, range of motion, foot position), injury and training history (via questionnaire), impact loading (via accelerometery) and running technique measures were collected at baseline. Runners were tracked for injury for one year via fortnightly check-ins. A binary logistic regression, (injury versus no injury), was performed for each variable univariably, and then adjusting for age, sex and mileage. A multivariable regression was also performed to evaluate the model’s discriminative ability.

**Results:**

Of the 225 runners included in the final analysis 52% experienced a running related injury. Injury history in the past year, less navicular drop, and measures of running technique (knee, hip, and pelvis kinematics) were associated with increased odds of injury (*p* < .05). The multivariable logistic regression model was statistically significant, χ^2^(11) = 56.45, *p* < .001, correctly classifying 74% of cases with a sensitivity and specificity of 72% and 76%, respectively. The area under the receiver operating characteristic curve was 0.79 (CI_95%_ = 0.73–0.85), demonstrating acceptable discriminative ability.

**Conclusions:**

This study found a number of clinical and running technique factors to be associated with prospective running related injuries among recreational runners. With the exception of injury history, the factors identified as being significantly associated with injury may be modifiable and therefore, could form the basis of interventions. Range of motion, spatiotemporal parameters and strength measures were not associated with injury and thus their utilisation in injury prevention practices should be reconsidered.

## Introduction

The high incidence of running related injuries (RRIs) [1,2] and their negative implications on both physical and mental health [3] underscores the importance of understanding the associated risk factors. The challenge in identifying risk factors is that they are multifactorial in nature [2]. Given that RRIs are caused by high load in excess of tissue capacity [4,5], risk factors generally relate to: (1) load (e.g. impact accelerations, ground reaction force), (2) factors affecting load (e.g. running technique, BMI, sex, age), and/or (3) the ability of biological tissue to tolerate load (e.g. previous injury history, clinical measures [strength measures, foot position, flexibility]).

The most common method of indirectly quantifying loading during running has been by assessing ground reaction forces (GRFs) via force plates. However, there are mixed findings on their association with injury [2,6] A limitation of GRF assessment is that it captures whole-body loading and therefore, does not assess segment-specific loading. Segmental measurement would be more appropriate because injuries are site-specific and because loading distribution throughout the body and across runners are not homogenous. Impact accelerometers, which indirectly assess segmental loading [7], are low-cost and easy to use, making them potentially more appropriate to a clinic-based setting and have shown good reliability across timepoints [8].

Factors that affect loading or the ability of the body to tolerate loading have also been the subject of RRI research. These range from high-resource, time-consuming measures of running technique using motion analysis systems [2], to low-cost, easily implementable clinical measures such as: range of motion (ROM) [9,10], foot positioning [10] and muscle strength [11,12]. Furthermore, training history [1], previous injury history [1,13], sex [2], age [14] and BMI [1] may also affect the load and tissue integrity.

Although studies have investigated RRIs, many have examined a small number of factors, potentially failing to account for important risk factors. Furthermore, many studies have utilised a retrospective approach comparing currently injured runners to healthy controls [15,16]. Therefore, differences identified between injured and uninjured runners may be from alterations due to pain or a consequence of the injury, preventing appropriate conclusions regarding the actual risk factors for RRIs. This necessitates the execution of prospective large-scale studies, which are arguably more time and resource intensive to run but can provide valuable information regarding risk factors preceding injuries, which may help us to identify appropriate preventative measures. Few studies have examined multiple factors related to RRI in a prospective manner [1,2]. Perhaps the largest is that of Messier et al. (2018) [2] who found greater knee stiffness, but not clinical measures, to be associated with greater odds of injury. However, they used GRFs to quantify loading, which do not reflect segmental loading (discussed above), and kinematic analysis was limited to the lower leg, despite kinematics further up the body being reported as potentially related to injury [17]. Therefore, examinations involving impact accelerometery and kinematics of the thorax, hip and pelvis are required.

This study aimed to prospectively examine the association between RRIs and demographics, injury history and training history, clinical measures, impact accelerations and running technique in a large cohort. A secondary aim was to evaluate the potential of these factors in screening for RRI. It was hypothesised that factors which indicate higher loading (e.g. greater impact accelerations) or reduced ability to dissipate loading (e.g. decreased foot arch, lower muscle strength, less sagittal knee flexion angle at initial contact, foot strike pattern) would be associated with the occurrence of injury.

## Materials and methods

### Protocol

Two hundred and seventy-four recreational runners were recruited from Dublin and its surrounding areas (from January—September 2018) for participation in this prospective study via national radio, social media advertisement, phone calls to running clubs and leaflet distribution at running events. Inclusion eligibility was as follows: recreational runners, between 18–65 years old, with no history of injury within the last three months(8). Participants were excluded if they participated in contact, team, or high impact sports, to limit the effects of injuries related to non-running activities. A recreational runner was defined as a person who ran a minimum of 10km per week, for at least six months prior to inclusion in the study [18]. Participants were excluded if they previously or currently participated at an international level. An *a priori* power analysis was conducted using G*Power (G*Power V3.1, Heinrich-Heine-Universität, Dusseldorf, Germany) to determine the minimum sample size for a logistic regression analysis in this exploratory prospective study [19]. To achieve 80% power for detecting an odds ratio of 1.7 at a significance criterion of α = .05, a total of *N* = 170 participants was required. Accounting for a potential attrition rate of 20% [1,2] a total of 204 participants was required.

**Fig 1:**
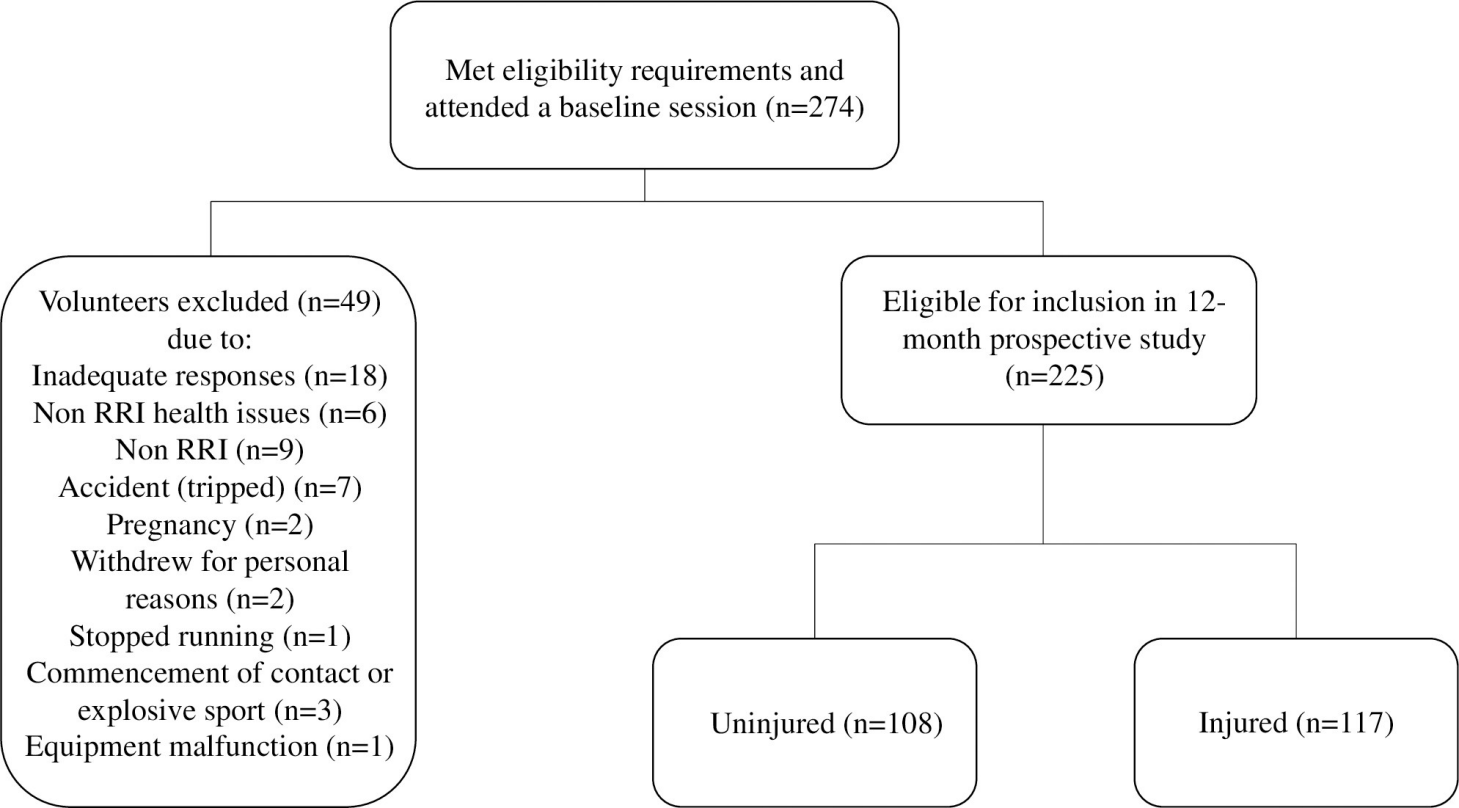
Flowchart of participants included in the study.

This trial was registered prior to recruitment (ClinicalTrials.gov Identifier: NCT03671395). Ethical approval was granted by Dublin City University Ethics Committee (DCUREC/2017/186). Participants were provided with a plain language statement form and screened for inclusion and exclusion criteria. Participants provided written consent to participate in the study. Eligible participants completed an online survey regarding their injury and training history prior to attending a baseline testing session, lasting approximately two hours. Fig 1 shows the flowchart of participants.

On the day of testing, participants completed a PAR-Q health clearance form and an informed consent form. The online survey responses were checked with the participant for accuracy.

### Clinical tests protocol

Anthropometric measurements and musculoskeletal clinical tests were performed for each participant by one investigator in line with the protocol of Dillon et al. (2021) [20]. Ankle dorsiflexion, hip extension, hip internal rotation, and external rotation range of motion were assessed for each leg and an average of three trials was used using a digital inclinometer. Foot position, navicular drop and foot posture index (FPI-6) were measured on each foot. The navicular drop is a measurement of the displacement of the navicular bone from a subtalar neutral sitting position to standing. The larger the displacement, the larger the collapse of the arch of the foot. The start and end test positions of the navicular bone were marked on a card, and the displacement was then measured with a ruler to the nearest millimetre. The Foot Posture Index is a static measurement of the foot on multiple planes (see Redmond et al. [21] for more information). This clinical tool rates six different measures of foot alignment, with higher scores indicative of a more pronated foot posture and lower values indicative of a more supinated foot. Manual isometric muscle testing using a hand-held dynamometer (J-Tech Commander Echo Wireless Muscle Testing Starter Kit, J-Tech Medical Industries, Midvale, UT, USA) (with and without stabilisation belt) was performed for the following: hip abduction, hip extension, plantarflexion, knee flexion and knee extension, with the maximum of three trials selected and normalised to moment arm and body mass. Participants were directed to use maximum effort and were given 15 second rest periods between bouts. Moderate to excellent intra-rater reliability was found for these measures (ICC = 0.83–0.99, lower–upper bound 95% CI = 0.53–0.99) [20].

### Running motion analysis protocol

A standardised dynamic lower-body warm-up was performed. Subsequently, participants performed a treadmill run (Runner-DTM2500, Flow Fitness, Amsterdam, Netherlands) wearing the footwear they normally use for running. They ran for 6 minutes at a speed of 9 km/hr for warm-up and treadmill familiarisation. Participants then ran for three minutes at a self-selected pace that best represents their typical training pace.

During the first minute of the self-selected treadmill run, kinematic data were collected using a 17-camera, 3D motion analysis system (Vicon Vantage, Oxford Metrics PLC, Oxford, United Kingdom) at a sampling rate of 200 Hz. Thirty-two reflective markers, 14 mm in diameter, were placed by one investigator (SD) on bony landmarks of the trunk, pelvis and lower limbs according to a Plug in Gait model (Vicon, Oxford Metrics PLC, Oxford, United Kingdom), with additional markers on the anterior aspect of the mid-tibia and mid-thigh bilaterally.

Peak acceleration (peak_accel_) and rate of acceleration (rate_accel_) were measured using three inertial measurement units, with two attached to the shank and one to the lower back (IMUs) (Shimmer3 IMU unit, Shimmer, Dublin Ireland). IMUs have previously been found to show high to excellent reliability over long and short term periods [8] and are a surrogate measure of loading [7,22]. IMUs (dimensions: 65 mm x 32 mm x 12 mm, weight: 31 gm, acceleration range: ± 16 g) were attached tightly bilaterally to each shank, 5 cm proximal to the medial malleolus, using Hypafix tape, and aligned with the long-axis of the shank. A single IMU (dimensions: 51 mm x 34 mm x 14 mm, weight: 23.6 gm, acceleration range: ±8 g) was secured tightly with a custom-made belt adhered to the skin using double sided sticky tape and another belt overlaying it. The negative y-axis aligned superiorly along the vertical midline of the S2 spinous process. This was secured further by tape and an elastic waistband in line with recommendations that wrapping is more representative of tibial accelerations [23]. The IMU triaxial accelerometer data were captured at 512 Hz. Sensors were calibrated using the Shimmer 9DOF Calibration Application.

### Injury tracking protocol

After this testing session, participants were encouraged to train as normal and RRIs were tracked prospectively for one year. Participants were emailed every 2 weeks enquiring about injuries and were also encouraged to contact researchers at the time of any injury. An RRI was defined in line with a consensus statement [24] as any muscle, bone, tendon or ligament pain in the lower back, hip, groin, thigh, leg, knee, foot, ankle and toe that caused the participant to stop or restrict their running. The pain must have persisted for at least 7 days or 3 consecutive scheduled training sessions or required the participant to consult a physician or other health care professional. All injuries were diagnosed by the researchers (SD (Chartered Physiotherapist) and AB (Certified Athletic Therapist). Where this was not possible, the diagnosis was confirmed via phone call. Severity was reported as minor, moderate, serious or long-term if 1–7 days, 8–28 days, 29 days-6 months or greater than 6 months was missed, respectively [25]. Additionally, if no days were missed this was recorded as a minor injury. Participants were removed if they did not respond to at least 80% of check ins.

### Data management and analysis

Clinical and anthropometric data (detailed in Table 1) were inputted into an Excel Sheet. Weight and height were used to calculate BMI. For the range of motion and navicular drop variables, the average of three trials was recorded. Additionally, using navicular drop values, participants were categorised into two groups, >10mm and <10mm, as measurements exceeding 10mm have previously been found to be related to injury [26]. FPI scores were used to allocate participants into one of three foot type categories: supinated (-1 to -12), neutral (0 to +5) and pronated (+6 to +12). Isometric muscle strength was normalised to moment arm (distance from the fulcrum to the application of force) and body mass. The maximum of three trials was recorded. Accelerometery data were processed using custom-written software (MATLAB R2018a) and were filtered using a 4th -order zero-lag Butterworth filter (60 Hz) [27]. A cubic spline was used to fill dropped packets, and the time series data were time-aligned via the Shimmer software (Shimmer Consensys, Ireland). With respect to the kinematic data, rigid body segments of the thorax, pelvis, thigh, shank and foot, and the joint angles (of the foot, knee, hip, thorax) between these segments in all three planes were defined by the Plug in Gait Model in Nexus 2 (Vicon, Oxford Metrics PLC, Oxford, UK). Filtering of Vicon marker trajectories was done using a fourth-order zero-lag Butterworth filter at 15 Hz. A cutoff frequency of 15 Hz was chosen by residual analysis [28]. To improve accuracy of the kinematic data, a number of approaches were undertaken [29]. Functional joints were calculated using the ‘OSSCA’ method. Hip joint centre and the functional knee axes were calculated using the symmetrical centre of rotation estimation (SCoRE) and the symmetrical axis of rotation approach (SARA), respectively. Soft tissue artefact was minimized using the optimal common shape technique (OCST). Stance data at discrete time points were extracted from 30 strides for analysis (peak angle, minimum angle, excursion angle, angle at initial contact, angle at toe-off). In relation to foot strike pattern, categories were determined in line with previous recommendations, with foot angle at initial contact over 8.0° representing rearfoot strike, less than -1.6° representing forefoot strike and between -1.6° to 8.0° indicating a midfoot strike [30]. The full list of included variables can be seen in Table 1.

**Table 1:** Variables examined within this study and entered into a univariable analysis.

Factors that quantify the magnitude of this load	Factors affecting load dissipation	Factors which capture the ability of tissue to tolerate load
Peak shank accelerations (g) Peak lower back accelerations (g) Shank rate of accelerations (g/s) Lower back rate of accelerations (g/s) Stride frequency (stride/min) Self-reported average running pace (km/hr) Self-reported 3 monthly mileage (km) Running experience (<10 years, 10+ years, reference is <10 years’ experience)	Spatiotemporal Parameters: Foot strike pattern (FFS, MFS, RFS, reference is RFS) Flight time (milliseconds) Stride length (metres) Contact time (milliseconds) Step time (milliseconds) Stance phase ankle, knee, hip, pelvis, trunk angles (degrees) [sagittal, frontal, transverse plane] at: Initial contact Toe-off Peak Minimum Excursion Age (years) BMI (kg/m) Sex (reference is female)	Navicular drop (mm) Navicular drop > 10 mm/< 10 mm (reference value is < 10 mm) Foot Posture Index (supinated, neutral, pronated, reference value neutral foot) Hip abduction strength (Nm/kg) Hip extension strength (Nm/kg) Plantarflexion strength (Nm/kg) Knee extension strength (Nm/kg) Knee flexion strength (Nm/kg) Knee to wall ROM (degrees) Hip extension ROM (degrees) Hip internal rotation ROM (degrees) Hip external rotation ROM (degrees) History of RRI < 1 year ago/>1 year ago (reference is RRI <1 year ago)

*Examined solely on sagittal plane.

Abbreviations: FFS- forefoot strike, MFS- midfoot strike, RFS- rearfoot strike.

Participants were first divided into prospectively injured and prospectively uninjured groups. Among injured runners, variables of interest were taken from the side of the body which was reported to have sustained the first injury. This was done for two reasons. Firstly, some participants experienced injuries on both sides of the body. Although values on both sides could have been averaged, it was hypothesised that the side of the first injury would most accurately reflect factors causative of injury. Secondly, it was hypothesised that subsequent injuries may be as a result of the first injury. To minimise the effects of limb dominance, the percentage of injuries sustained on the dominant and non-dominant sides was calculated. The same proportion of dominant and non-dominant sides were selected at random for the uninjured runners. Where first injuries were bilateral or central in nature (e.g. central low back pain), the dominant side was used.

The variables assessed are outlined in Table 1. Five categorical variables were included: sex, injury history, running experience, foot strike pattern and navicular drop (greater than or less than 10mm). To assess the primary aim, a univariable binomial regression for each variable was performed, with significance set at p < .05. Since sex [2], age [14] and mileage [1] may be associated with injury, these were included as covariates and both the adjusted and unadjusted results reported. To assess the secondary aim, variables with a p value < .25 were entered into a multivariable logistic regression [31]. Linearity of the continuous variables with respect to the logit of the dependent variable was assessed via the Box-Tidwell procedure [32]. A Bonferroni correction was applied using all terms in the model resulting in statistical significance being accepted when p < .00172. Based on this assessment, all continuous independent variables were found to be linearly related to the logit of the dependent variable. Multicollinearity was assessed using Spearmon’s Rho Correlations (S3 Table). Where variables were correlated (>0.7) [33], the variable with the highest statistical significance was used. Imputing missing variables was achieved by utilising the: clinical, kinematic, anthropometric and demographic variables, along with the training history [34]. Numeric data were first scaled to unit variance and zero mean, while the categorical data were dummy encoded. Data were then imputed using multivariable imputation by chained equations and a Bayesian ridge regression approach [34]. All variables with a univariate association p < .25 were included in a binomial regression using backward stepwise selection logistic regression. P < .05 was considered statistically significant (SPSS Statistics version 27, IBM Corporation, Armonk, New York, USA). Odds ratios and confidence intervals were reported for each variable. With respect to continuous variables, odds ratios > 1 indicate that as the predictor variable increases injury is more likely to occur. Odds ratios < 1 indicate that as the predictor variable increases injury is less likely to occur. For categorical predictors, the odds ratio compares the odds of injury between a reference category and a predictor category, with OR >1 indicating that that injury was more likely to occur in those falling into the predictor category.

A receiver operating characteristic (ROC) curve was performed to determine the model’s discriminatory ability. The area under the curve values were interpreted with: <0.5, >0.5 to 0.7, >0.7 to 0.8, > 0.8 to 0.9, > 0.9 representing no, poor, acceptable, excellent and outstanding discrimination, respectively [35].

To assess the usefulness of factors with high relevance to clinical practice, an additional multivariable binomial regression was run solely using factors considered cost and time efficient: clinical measures [foot position, muscle strength, flexibility], injury history, training history and demographics. This was undertaken using the same procedure outlined above.

## Results

Of the 274 runners entering the study, 225 runners (82%) remained in the study to follow up (84 females, 141 males, weight = 74.7 kg, height = 1.73m, BMI = 24.0 kg/m^2^, age = 43.5 years). Reasons for exclusion are detailed in Fig 1. Over the 1-year period, 52% (n = 117) reported at least one RRI. The location (S1 Table), diagnoses (S2 Table) and severity (Table 2) of injury are reported, with injured runners missing an average of 56 days due to RRI. Calf strain (15%), followed by Achilles tendon injury (11%) and plantar fasciopathy (9%) constituted the largest proportion of injuries (S2 Table).

**Table 2:** Severity of injury[25].

Severity of injury	Number of first injuries of this severity (percentage)
No days missed	12 (10%)
Minor (1–7 days missed)	12 (10%)
Moderate (8–28 days missed)	43 (37%)
Serious (29 days- 6 months missed)	44 (38%)
Long term (>6 months missed)	6 (5%)

Demographic and anthropometric characteristics of participants are detailed in Table 3. There were no differences in running speed between the injured and uninjured runners (mean difference = -0.006 km/hr, CI_95%_ = -0.40–0.39, t (223) = -.029, p = .977).

**Table 3:** Results of the univariable regression for each demographic, anthropometric, training history and injury history variables.

Variable	Uninjured	Injured	Unadjusted				Adjusted*			
	Mean ± SD	Mean ± SD	Sig.	OR	95% C.I. for OR		Sig.	OR	95% C.I. for OR	
Demographics and anthropometrics					Lower	Upper			Lower	Upper
Age (years)	43.6 ± 9.3	43.4 ± 8.4	0.888	1.00	0.97	1.03				
BMI (kg/m^2^)	24.2 ± 2.9	23.7 ± 2.9	0.188	0.94	0.86	1.03	0.094	0.92	0.83	1.02
Female sex (reference is male)	41 females (38%)	43 females (37%)	0.851	0.95	0.55	1.63				
**Training and injury history**										
History of RRI < 1 year ago	38 (35%)	57 (49%)	0.041	1.75	1.02	2.99	0.05	1.72	1.00	2.95
Self-reported weekly mileage (km/week)	435 ± 255	410 ± 233	0.443	1.00	2.00	1.00				
Self-reported average running pace (km/hr)	11.3 ± 1.9	11.5 ± 1.6	0.289	1.09	0.93	1.27	0.222	1.11	0.94	1.31
Running experience > 10 years (reference is <10 years’ experience)	27 (25%)	24 (21%)	0.422	0.77	0.41	1.45	0.383	0.76	0.40	1.42

Abbreviated terms: SD = standard deviation, C.I. = confidence interval, Sig = significance level, OR = odds ratio. Adjusted for sex, age and mileage.

### Univariable analysis

**Fig 2:**
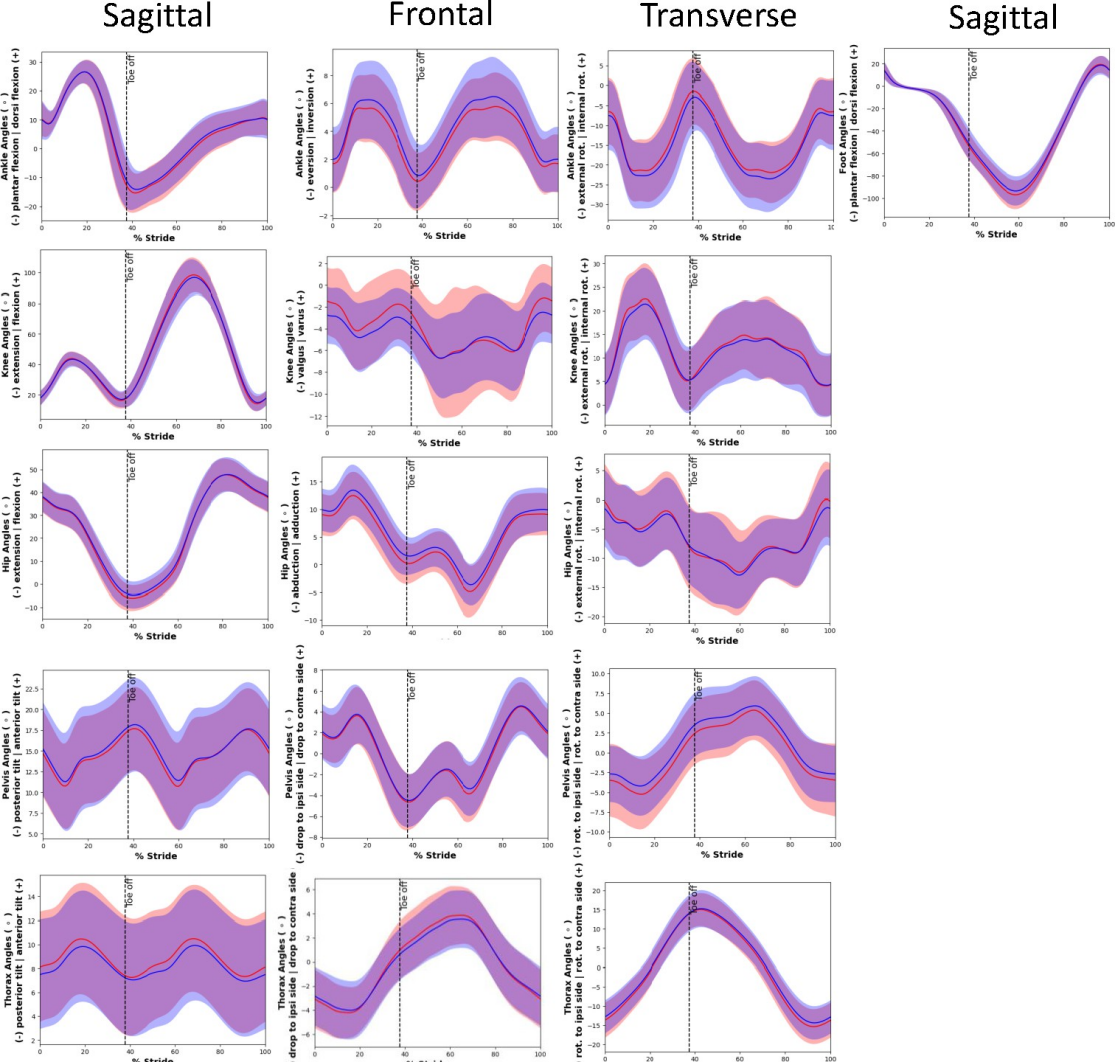
Graphical representation of kinematics during the entire gait cycle. The red line represents the injured group with the blue representing the uninjured group. The coloured bands represent the standard deviation of each group.

The univariable analysis (Table 4) revealed the following factors were significantly (p < .05) associated with the development of an RRI: clinical measures (less navicular drop), previous injury history < 1 year ago, and running technique (greater knee internal-external rotation excursion, less minimum knee valgus, less hip adduction at toe-off, less transverse plane peak pelvis contralateral rotation, less transverse plane pelvis contralateral rotation at toe-off, less knee valgus at initial contact, less peak knee valgus, less knee valgus at toe-off, (Fig 2). After adjusting for age, weekly mileage and sex, the following were additionally statistically significant and associated with RRI: navicular drop <10 mm, greater transverse plane peak pelvis ipsilateral rotation, greater knee valgus-varus excursion, less minimum hip adduction. A complete report of the univariable analysis is available in S4 Table. For clarity, when two significant variables were highly correlated (S3 Table), terms were grouped for the discussion.

**Table 4:** Variables that were significantly associated with injury in the univariable regression.

Variable	Uninjured	Injured	Unadjusted				Adjusted*			
	Mean ± SD	Mean ± SD	Sig.	OR	95% C.I. for OR		Sig.	OR	95% C.I. for OR	
Transverse plane pelvis contralateral rotation at toe-off (°)	3.6 ± 4.1	2.3 ± 3.9	0.018	0.92	0.86	0.99	0.014	0.92	0.85	0.98
Transverse plane peak pelvis contralateral rotation (°)	3.8 ± 3.7	2.8 ± 3.7	0.029	0.92	0.86	0.99	0.024	0.92	0.85	0.99
Transverse plane minimum pelvis contralateral rotation (°)	-5.3 ± 3.6	-6.4 ± 4.2	0.056	0.94	0.87	1.00	0.042	0.93	0.87	1.00
Hip adduction at toe-off (°)	1.1 ± 3.4	0.0 ± 3.7	0.022	0.92	0.85	0.99	0.024	0.92	0.85	0.99
Minimum hip adduction (°)	0.9 ± 3.4	-0.2 ± 3.7	0.057	0.94	0.88	1.00	0.023	0.91	0.85	0.99
Knee varus at initial contact (°)	-2.9 ± 2.8	-1.5 ± 3.1	0.001	1.19	1.08	1.32	0.001	1.20	1.08	1.33
Peak knee varus (°)	-2.1 ± 2.7	-0.8 ± 3.1	0.003	1.16	1.05	1.28	0.003	1.16	1.05	1.28
Knee varus at toe-off (°)	-3.9 ± 33.0	-2.7 ± 3.1	0.004	1.15	1.05	1.26	0.004	1.15	1.04	1.26
Minimum knee varus (°)	-5.9 ± 2.9	-5.0 ± 3.5	0.032	1.10	1.01	1.19	0.038	1.10	1.01	1.19
Knee varus valgus excursion (°)	3.9 ± 1.6	4.3 ± 1.7	0.060	1.17	0.99	1.38	0.049	1.18	1.00	1.40
Knee internal rotation external rotation excursion (°)	20.9 ± 4.5	22.4 ± 5.4	0.024	1.07	1.01	1.13	0.024	1.07	1.01	1.13
Navicular drop (mm)	9.0 ± 3.3	7.9 ± 2.9	0.005	0.88	0.81	0.96	0.004	0.87	0.80	0.96
Navicular Drop > 10 mm (Reference is navicular drop <10 mm)	40 (37%)	30 (26%)	0.066	0.59	0.33	1.04	0.05	0.56	0.31	1.00
History of RRI < 1 year ago (reference is injury > 1 year ago/never injured)	38 (35%)	57 (49%)	0.041	1.75	1.02	2.99	0.05	1.72	1.00	2.95

Abbreviated terms: SD = standard deviation, OR = odds ratio, C.I. = confidence interval. The following denote the direction of the moment:Knee varus (positive), knee valgus (negative), knee internal rotation (positive), knee external rotation (negative), hip adduction (positive), hip abduction (negative), pelvis anterior tilt (positive), pelvis rotation to contralateral side (positive), pelvis rotation to ipsilateral side (negative).

* Adjusted for sex, age and mileage.

### Multivariable analysis

Regarding the multivariable analysis, only 11 variables remained in the final model, with eight being statistically significant (p < .05): previous injury history < 1 year ago, less navicular drop, lower BMI, less knee flexion strength, less knee valgus at initial contact, greater hip internal-external rotation excursion, greater thorax contralateral-ipsilateral side flexion excursion and less pelvis internal rotation at toe-off (Table 5). The multivariable logistic regression model was statistically significant, χ^2^(11) = 56.45, *p* < .001, correctly classifying 74% of cases. Sensitivity was 72% and specificity was 76%, the positive predictive value was 75% and the negative predictive value was 74%. The area under the ROC curve was 0.79 (CI_95%_ = 0.73–0.85), demonstrating acceptable discriminative ability between injured and uninjured runners.

**Table 5:** Results of the multivariable regression.

Variable	Uninjured	Injured	Sig.	OR	95% C.I. for OR	
	Mean ± SD	Mean ± SD			Lower	Upper
Thorax contralateral-ipsilateral side flexion excursion (°)	5.1 ± 1.7	5.6 ± 2.2	0.04	1.18	1.01	1.39
Transverse plane pelvis contralateral drop at toe-off	2.2 ± 2.7	1.9 ± 2.3	0.00	0.86	0.79	0.94
Hip internal rotation external rotation excursion (°)	10.8 ± 3.8	11.7 ± 3.8	0.04	1.18	1.01	1.39
Hip adduction at initial contact (°)	10.1 ± 4.0	9.1 ± 3.8	0.09	0.92	0.83	1.01
Hip adduction at toe-off (°)	1.1 ± 3.4	0.0 ± 3.7	0.10	1.10	0.98	1.23
Knee varus at initial contact (°)	-2.9 ± 2.6	-1.5 ± 3.1	0.00	1.24	1.08	1.41
Navicular drop (mm)	9.0 ± 3.3	7.9 ± 2.9	0.00	0.84	0.76	0.93
Knee flexion strength (Nm/kg)	0.99 ± 0.28	0.93 ± 0.25	0.01	0.17	0.05	0.63
BMI (kg/m^2^)	24.2 ± 2.9	23.7 ± 2.9	0.02	0.86	0.76	0.97
History of RRI < 1 year ago (reference is injured >1 year ago)	38 (35%)	57 (49%)	0.03	2.03	1.08	3.78
Female sex (reference is male)	41 females (38%)	43 females (37%)	0.09	0.50	0.22	1.13

Abbreviations: SD = standard deviation, CI = confidence interval, OR = odds ratio. The multivariable logistic regression model was statistically significant, χ^2^(11) = 56.45, p < .001. The model correctly classified 74% of cases. Sensitivity was 72%, specificity was 76%. The area under the ROC curve was 0.79 (CI_95%_ = 0.73–0.85).

A further analysis was conducted investigating the relationship between prospective RRI and the factors considered relatively cost and time efficient to assess (clinical measures [foot position, muscle strength, flexibility], injury history, training history, demographics and anthropometrics) (Table 6). History of injury in the past year, less navicular drop, and lower hip abduction strength significantly contributed to the model. The model was statistically significant, χ^2^(5) = 21.38, p = .001 and correctly classified 63% of cases. Sensitivity was 59%, specificity was 66%, positive predictive value was 64% and negative predictive value was 62%. The area under the ROC curve was 0.67 (CI_95%_ = 0.60–0.74), demonstrating poor discriminative ability between injured and uninjured runners.

**Table 6:** Results of the multivariable regression for clinical factors.

Variable	Uninjured	Injured	Sig.	OR	95% C. I. for OR	
	Mean ± SD	Mean ± SD			Lower	Upper
Navicular drop (mm)	9.0 ± 3.3	7.9 ± 2.9	0.01	0.88	0.80	0.96
History of RRI < 1 year ago (reference is injured >1 year ago)	38 (35%)	57 (49%)	0.01	2.24	1.25	4.02
Hip abduction strength (Nm/kg)	1.70 ± 0.32	1.64 ± 0.30	0.01	0.28	0.10	0.76
BMI (kg/m^2^)	24.2 ± 2.9	23.7 ± 2.9	0.05	0.90	0.80	1.00
Female sex (reference is male)	41 females (38%)	43 females (37%)	0.07	0.55	0.28	1.06

Abbreviations: SD = standard deviation, Sig = significant, CI = confidence interval, OR = odds ratio. The model was statistically significant, χ2(5) = 21.38, p = .001. The model correctly classified 63% of cases. Sensitivity was 59%, specificity was 66%. The area under the ROC curve was 0.67 (CI_95%_ = 0.60–0.74).

## Discussion

Where possible, the findings of the present study were compared to previous prospective studies investigating general RRIs. This was done as the cause-effect response to injury is unclear in retrospective studies, with the possibility of injuries producing compensatory changes that are directly opposite to true causative factors. This study aimed to investigate factors associated with RRI among recreational runners. Largely, our results were not in line with the hypothesis that measures of loading (impact acceleration) or factors affecting the dissipation of load (muscle strength, knee flexion angle at initial contact, foot strike pattern) would be associated with injury.

### Injury incidence

The one-year injury incidence of 52% is similar to other studies [1,13]. The calf constituted the highest proportion of injuries, as found in previous research [1]. However, the knee was only the third most reported injury site, despite it being frequently cited as the most common [36]. In terms of injury diagnosis, calf strain (15%), followed by Achilles tendon injury (11%) constituted the largest proportion of injuries. In their study of injury diagnoses within a 24-week tracking period, Mulvad et al. (2018) [37] found medial tibial stress syndrome, followed by Achilles tendon injury to be the most common diagnosis in recreational runners. The difference from our study may be explained by injuries being grouped differently. For example, what we classified as “calf injuries” were subdivided into “soleus injuries” and “gastrocnemius injuries” by Mulvad et al. (2018) [37]. Had these injuries been grouped, as in our study, calf injuries would have been the second most common injury, pointing to the need for standardised classification and reporting of injuries. The comparatively small proportion of MTSS injuries may be reflective of the greater weighting of females in the study by Mulvad et al., (2018) [37], as previous research suggests that female sex is a risk factor for this injury [38]. The majority of injuries were classified as “serious”, indicating that they lasted 28 days-6 months; the average number of missed days was similar to previous research [37].

### Univariable analysis

#### Anthropometric and demographic factors

The associations between RRI and anthropometric and demographic factors such as BMI, age and sex have been debated within the literature. Greater BMI has been theorised to be associated with increased risk of injury by placing increased load per step; although its association to RRI has been debated [1,13]. Older age is suggested to be related to increased risk of RRI [14], possibly due to changes in running technique [39], and decreased muscle strength [39]. However, research is mixed [13,14]. In our study, age, BMI and sex were not found to be associated with RRI. A comparable proportion of males and females became injured during the one-year tracking period, a finding echoed elsewhere [13].

#### Injury and training history

An injury in the past year (injury history) was found to be associated with a prospective injury in both the multivariable and univariable analyses, increasing odds of injury by over two times. This is in line with a previous systematic review [40]. There are two primary explanations for this relationship. Firstly, previously injured tissues may not have adequately healed [41]. Secondly, injury-related pain may lead to an alteration in running technique [41], which may persist following return to sport. This alteration may overload biological structures, precipitating future injury. In our study, while injury in the previous year increased the odds of injury in the final multivariable model, a previous injury of greater than one year did not. This supports the suggestion that with a shorter time frame since injury, runners are more vulnerable to re-injury [42]. This indicates that athletes, clinicians and coaches should be particularly cognisant of runners with an injury within the preceding year.

No association was found between RRI and either measure of training history (self-reported pace, average weekly mileage in past three months). Regarding pace, our findings were in line with previous studies investigating RRIs [2,43]. However, the association between RRI and weekly mileage has been conflicting [1,2]. Our findings provide evidence to indicate that recall of weekly mileage may not provide clinicians with information useful for indicating who will sustain an injury. However, given the theoretical link between increased load and injury [4,5], other measures of capturing volume of loading, such as strides/session, should be explored.

Spatiotemporal parameters such as stride length, flight time, step time, stance time and stride rate were not associated with injury. Spatiotemporal parameters are relatively easily measurable and have been postulated to be related to RRI. A common suggestion is that manipulation of these factors can reduce injury risk via load reduction [44]. Most research investigating the association between RRI and spatiotemporal parameters is retrospective, therefore, this study adds important information to this area. To our knowledge, just one prospective study has investigated the association between general RRI and a number of spatiotemporal parameters during running [1], finding significantly greater flight time and lower step rate among injured compared to uninjured runners. However, this was based on a sample size of 31 runners and this finding only pertained to females. Adding credence to our lack of significant findings, a systematic review by Brindle *et al*. [45] found no difference in mean stride time, stance time, cadence, and stride length between uninjured and injured runners via a meta-analysis.

#### Clinical measures

Clinical measures such as strength [12], range of motion [10,13] and foot positioning [10] have widely been hypothesised to be associated with injury, with modification via strengthening, stretching and orthoses suggested in injury intervention methods [46]. We found just one clinical measure, the navicular drop test, to be associated with injury. Unlike static measures such as Foot Posture Index, which was not found to be univariately related to injury in our study, navicular drop captures the dynamic mobility of the foot and is thought to represent the movement of the medial longitudinal arch [47]. It is also suggested to be related, although weakly, to rearfoot eversion [48], making it potentially useful for clinicians in lieu of biomechanical motion analysis equipment. Our results showed that with less navicular drop, the odds of future RRI increased. Our results also indicated that navicular drop < 10 mm, which is a previously used cut-off point [26,49], increased odds of injury by two times. Largely, findings from studies investigating the relationship between general RRIs and navicular drop on a continuous level [10,43] or using cut-off points [26,49] have been mixed. It should be noted, that similar to our study, Dudley et al. (2017) [43] reported mean navicular drop of injured participants to be 17% less than that of uninjured runners, however, this did not reach significance. Lack of significance may have been related to the small sample size (n = 31). An explanation of our findings may be that uninjured runners have a more flexible foot, with increased capability in absorbing loads during stance [50]. However, the mean values of navicular drop test observed in our study would place the uninjured runners in the “pronated” category and the injured runners in the “neutral” category [51]. Therefore, although injured runners may not have as much arch collapse as the uninjured group, they do demonstrate some flexibility of the foot. Secondly, a minimum detectable change of 1.70–2.22 mm has previously been reported [47] and differences between our groups does not exceed this. Therefore, from a clinical perspective this finding should be interpreted cautiously, although these results indicate that clinicians should reconsider running injury prevention and treatment techniques aimed at correcting “over- pronation”. There also did not appear to be an association between foot eversion and the occurrence of a RRI, contrary to the traditional belief that navicular drop is linked to eversion movement.

Lower strength has been suggested to be associated with RRI, with strengthening a target of injury prevention interventions [46]. However, research surrounding the association between prospective RRIs and strength has largely been inconsistent [11,12]. The present study indicates that isometric strength in a fixed position is not associated with RRI. It should be noted that running also requires concentric and eccentric muscle action [52] through a range of movement, which may not have been captured by the isometric testing protocol employed in our study. In addition, it is possible that maximum strength values were not captured, as they may be dependent on participant motivation. ROM has been inconsistently linked to RRI [9,10], with some authors suggesting that low ROM places excessive stress on joint [10] and others suggesting that high ROM increases demands on muscles to stabilise during movement [53]. Our large-scale study indicates that ROM values have very limited value in understanding the aetiology of RRI. This may be because the ranges of motion exhibited in these clinical tests are greater than those utilised during running, indicating that running is unlikely to produce strain-related injuries.

#### Running technique

**Fig 3:**
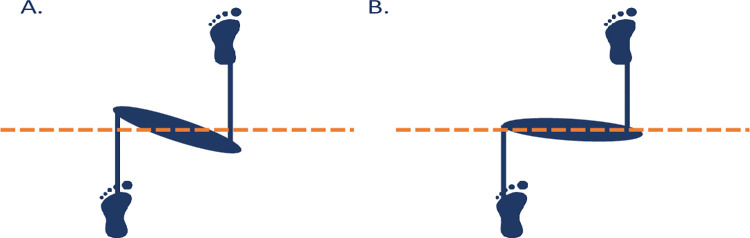
Transverse plane rotation of the pelvis at toe-off. The left leg is the stance leg at toe-off. A. demonstrates greater contralateral pelvic rotation as seen in the uninjured runners, with B. demonstrating less peak contralateral pelvic rotation, as seen in the injured runners.

Significant associations between the risk of RRIs and pelvis, hip and knee kinematics were found. Stance phase transverse plane pelvis rotation was found to be associated with RRI (Figs 2 and 3). During running, the pelvis rotates contralaterally and ipsilaterally in the transverse plane (whereby the anterior aspect of the pelvis rotates towards the swing and stance legs, respectively). Although the important role of transverse plane pelvic rotation in running for performance is well recognised [54], to the best of our knowledge, it has not been previously studied, either prospectively or retrospectively, in relation to RRIs. At initial contact the pelvis is in slight ipsilateral transverse plane rotation and this increases until midway through stance (Fig 3) [54]. During terminal stance and approaching toe-off the pelvis begins to contralaterally rotate. In our study, less peak pelvic contralateral rotation during stance was associated with increased odds of injury. During straight-line running, the balance of the angular momentum between the upper and lower body about the vertical axis must be maintained [55]. This is controlled by the interaction of movements of the head, arms, trunk, pelvis and legs in the transverse plane and the vertical free moment produced at the foot. The vertical free moment is the moment of force produced due to the friction between the foot and the ground during stance [56]. While it is not clear which is cause and which is effect, less pelvic contralateral rotation is reflective of higher vertical free moments at the foot, which are related to an increase in lower limb tortional stress [55]. An increase in torsional stress has been linked to injuries such as tibial stress fractures [56] and PFPS [55].

In the frontal plane, less hip adduction was found to be associated with injury. To our knowledge, frontal plane hip motion has only been examined in one prospective study with respect to general RRI, finding no association to exist [43]. However, Dudley *et al*. [43] investigated collegiate cross-country runners who may have distinctive injury risk factors. Our finding is in line with some previous retrospective studies examining specific injuries [16,17,57]. Primarily, explanations connecting less peak adduction to injury have focused on iliotibial band syndrome, unclear suggestions as to its aetiology [57]. Specifically the association between less hip adduction at toe-off and *general* RRIs may be explained by two reasons- less transverse plane contralateral pelvic rotation (discussed above) and/or increased trunk lateral flexion over the stance limb as a result of weak hip stabilisers [16,17]. However, our data did not find side flexion or hip abduction strength to be associated univariately with injury.

At the knee, less knee valgus and greater valgus-varus excursion were found to be univariately associated with increased odds of injury. The prevailing theory relating knee motion to injury suggests that extreme varus and valgus knee positions increase load bearing on the knee medially and laterally, respectively [58]. Over time, high patellofemoral stress overload the articular cartilage and subchondral bone, resulting in injury [59]. However, previous research investigating peak knee varus during the stance phase of running has been limited, finding no difference between those with and without general prospective RRIs [43]. In our study, it is in fact *less* peak valgus and *less* valgus at initial contact that is associated with injury. However, when considering the entire stance phase, injured runners displayed greater frontal excursion at the knee. Therefore, it is possible that greater frontal plane motion at the knee during stance signifies a lack of control of the knee, potentially causing increased pressure on both the medial and lateral knee. Greater knee excursion during stance, despite the similar stance time between injured and uninjured runners, may also indicate that the rate of loading on knee structures during stance was greater among injured runners. Although this has not been previously investigated during running in relation to general RRIs, in a study of team sport athletes, those displaying large frontal knee motion angles during a single leg squat were 2.7 times more likely to sustain a lower extremity injury [60]. This indicates that frontal joint excursion, rather than peak angles, is important in relation to RRIs.

In the transverse plane, our study found that greater knee internal-external rotation excursion was univariately associated with increased odds of RRI. Only one RRI study has previously investigated knee rotation excursion, finding no association in their retrospective cohort study involving currently injured runners with PFPS [61]. Greater knee rotation excursion may have lead to increased torsional loads on knee and thigh structures such as the iliotibial band (ITB) [17] and greater patellofemoral contact pressures on facets of the patella [62].

Our study found no sagittal plane running technique factors to be associated with prospective injury. This is very important given the preponderance of research studies and clinical examinations that focus predominantly or exclusively on sagittal plane motion. Notably, rearfoot striking has frequently been theorised to relate to injury via greater impact loading magnitudes [63] and loading rates [64]. However, even results from this research are conflicting [55], and limited by the dominance of retrospective research [64]. Our research indicates that no association existed between foot strike and injury, in line with a recent systematic review [65]. However, its relationship to specific injuries should be considered further due to the associations between foot strike patterns and structure specific loading, such as between rearfoot strike pattern and increase in knee joint stress [63] and between forefoot strike pattern and increase in Achilles tendon force [63].

Similarly, knee flexion has been hypothesised to be a cause of general RRIs, due to increase in contact forces with knee extension [66]; but few studies have examined this [2]. During gait, knee flexion aids in shock absorption at initial contact and throughout stance [66]. Our findings add weight to the existing evidence [2] that there is no association between general prospective RRIs and either peak knee flexion or knee flexion at initial contact.

#### Loading during running

Neither peak nor rate of impact acceleration at the shank and sacrum were associated with injury in this study. Previous research investigating impact accelerations and injury has been limited and conflicting, with just one study examining impact accelerations at the back among 76 runner [1]. Therefore, the present prospective study provides the strongest evidence to date that impact accelerations assessed at a single time point when tested on a treadmill do not significantly affect the odds of sustaining an RRI. Although there are a number of reasons to inform the hypothesis that loading in excess of tissues capabilities would be related to injury [4], our study may have found no such relationship to exist for three reasons. First, the magnitude of impact accelerations may not in isolation distinguish between injured and uninjured runners, but a combination of loading and accurate collection of training volume could be necessary to determine cumulative loading [5]. Second, impact accelerations were captured on a treadmill and therefore may not be representative of typical running surfaces. Third, it may not be excess loading, but lower tissue strength (which may include decreased contractile strength, decreased morphological strength, inadequate training adaptation in response to loading) among injured runners that make them susceptible to injury.

### Multivariable analysis

The multivariate analysis identified a significant model containing eight features. Less navicular drop, injury history <1 year ago, lower knee flexion strength and stance phase variables (i.e. greater thorax contralateral-ipsilateral side flexion, greater hip internal-external rotation excursion, greater knee varus valgus excursion, less pelvic contralateral rotation at toe-off and less knee valgus at initial contact) all significantly contributed to greater odds of RRI. While the area under the ROC curve indicates an acceptable level of discrimination (74% accuracy), the use of this information to screen for RRI has a number of practical limitations. Firstly, given the large number of factors which contribute to the model and the time and effort that would be required to identify these factors, using this as a screening tool may not be feasible. Secondly, almost 30% of runners who would become injured would not be identified and therefore would not receive an intervention. Issues with sensitivity of screening tools has been highlighted as a challenge in injury screening programmes *per se* previously [67]. Lastly, cut-off points would need to be established to make this viable as an injury screening approach. However, although these three points suggest challenges in relation to injury *screening*, the identified risk factors of RRIs provide the foundation for the design of intervention programmes that can be undertaken by all runners. Effective programmes are currently lacking for running, but should be implemented and tested for efficacy, as has been done in in other sports (e.g. soccer [68]).

Similarly, the additional analysis undertaken to determine the association between RRI and factors easily measurable by clinicians (clinical measures, training history, anthropometrics and demographics), showed limited discriminative ability.

### Clinical implications of research

This study found a number of factors to be associated with increased odds of general RRIs, the strongest of which was previous injury < 1 year ago. This may indicate that, following injury, some runners have not regained original tissue strength or that they have alterations in technique that increase their vulnerability to injury. We also found that running technique is related to injury. Running technique is amenable to change via running retraining protocols [69]. Intervention strategies targeting neuromuscular and technique-based interventions should be developed and tested for efficacy in the same way that FIFA 11 [68] and similar approaches have been developed in other sports. Our study provides important information for general injury prevention strategies for runners. Although the developed model could correctly classify 74% of cases and showed acceptable discrimination between injured and uninjured runners, given the large number of variables that contributed to this model and the time-consuming nature of measuring each of these variables, the feasibility of using all of these measures in practice is questionable. Moreover, it should be noted, that although the association between some kinematic variables and injury were statistically significant, the mean differences between the groups were very small. In terms of the clinical relevance, it is possible that these angles could be smaller than that which could be reliably detected by clinicians using 2D analysis [70], which is more widely available than 3D motion capture.

### Limitations

This study a number of limitations. Firstly, all risk factors for injury were measured at a single time point. However, it is not known whether these factors remained consistent between the initial baseline testing and the point of injury. Secondly, while there is a clear and significant value in identifying risk factors for *general* RRIs as a whole, it is possible that specific injuries may have different or perhaps conflicting injury risk factors. Some injury diagnoses were carried out via phone call. While this would not affect allocation of participants into injured or uninjured groups, this method of diagnosis could have led to inaccuracies in reporting of diagnoses. Future studies should employ a similar design as the present study, but with analysis of specific injuries. Pooling of data across research centres may facilitate this, as large numbers of runners would be required to appropriately analyse specific injuries. Thirdly, the running technique and impact loading analysis was performed on a treadmill. Although this was advantageous for simultaneous collection of running technique and impact loading, and increasing the number of strides examined, both measures may be affected by surface. Therefore, these results may not be ecologically valid for runners who typically do not train on a treadmill. Furthermore, although an effort was made to adjust for training volume, this was captured at one time point and may have changed over the course of the study. This study included runners injured within the previous year (although not in the last three months). In light of the fact that previous injury was found within this study, and others [1,13] to be associated with future injury, it is possible that previous injury may have altered the measured risk factors within these studies. Therefore, future studies should consider exclusively studying runners without a history of injury to ensure that differences between groups are not an artefact of previous injury. Finally, this study primarily adopted a biomechanical approach to injury. However, psychosocial variables such as personality, sleep and stress may contribute to the development of injuries or could interact with biomechanical factors to predispose and athlete to injury [71,72]. Future research should consider adopting a combined biopsychosocial approach to RRI risk factors.

## Conclusions

This large-scale prospective study investigated the association between general RRIs and anthropometrics, demographics, training history, injury history, clinical measures, impact accelerations and running technique. Of the clinical factors, history of injury in the past year and less navicular drop were univariately associated with increased odds of injury. In terms of stance-based measures of running technique, knee, pelvis and hip motion in the frontal and transverse planes were associated with increased odds of injury. Anthropometrics, demographics, strength, ROM and measures of impact acceleration were not significantly associated with injury. This study emphasises the multifactorial nature of running related injuries and finds that factors remain outstanding that contribute to RRI development. Although these factors had a 74% accuracy in discriminating between injured and uninjured; the use of these variables as screening tests may have limited value. However, these factors could form the basis for the design of much-needed intervention programmes, which should be subsequently investigated for efficacy. The identification of clinically accessible measures sufficiently able to identify risk factors remains challenging.

## Supporting information

S1 Table Locations of first running related injury. (DOCX)

S2 Table Diagnoses of first running related injury. (DOCX)

S3 Table Results of the correlation analysis. (DOCX)

S4 Table Results of the univariable analysis. (DOCX)
