# Supplementary material for: Running towards injury? A prospective investigation of factors associated with running injuries
Source: PLoS One. 2023 Aug 17;18(8):e0288814. doi: 10.1371/journal.pone.0288814 (PMC10434952; doi:10.1371/journal.pone.0288814)
Supplement: S1 Table — (DOCX) [file pone.0288814.s001.docx]

S1 Table. Locations of first running related injury.

| Location of injury | Number of first injuries at this location (percentage) | Males | Females |
| --- | --- | --- | --- |
| Calf | 31 (26%) | 18 (24%) | 13 (30%) |
| Foot | 23 (20%) | 15 (20%) | 8 (19%) |
| Knee | 17 (15%) | 13 (18%) | 4 (9%) |
| Buttocks | 12 (10%) | 6 (8%) | 6 (14%) |
| Thigh | 11 (9%) | 6 (8%) | 5 (12%) |
| Lower back | 9 (9%) | 8 (11%) | 1 (2%) |
| Shin | 9 (8%) | 5 (7%) | 4 (9%) |
| Hip | 5 (4%) | 3 (4%) | 2 (5%) |
