# Supplementary material for: Running towards injury? A prospective investigation of factors associated with running injuries
Source: PLoS One. 2023 Aug 17;18(8):e0288814. doi: 10.1371/journal.pone.0288814 (PMC10434952; doi:10.1371/journal.pone.0288814)
Supplement: S2 Table — (DOCX) [file pone.0288814.s002.docx]

S2 Table. Diagnoses of first running related injury.

| Diagnosis of injury | Number of first injuries with diagnosis (percentage) | Number of males with first injury (percentage) | Number of females with first injury (percentage) | Location of injury |
| --- | --- | --- | --- | --- |
| Achilles tendon pain | 18 (15%) | 8 (11%) | 10 (23%) | Calf |
| Calf strain | 13 (11%) | 10 (14%) | 3 (7%) | Calf |
| Plantar fasciopathy | 11 (9%) | 8 (11%) | 3 (7%) | Foot |
| Lower limb stress fracture | 10 (9%) | 7 (9%) | 3 (7%) | Various locations |
| Patellofemoral pain syndrome | 9 (8%) | 7 (9%) | 2 (5%) | Knee |
| Piriformis syndrome | 6 (5%) | 4 (5%) | 2 (5%) | Buttocks |
| Non-specific low back pain | 4 (3%) | 3 (4%) | 1 (2%) | Low back |
| Medial tibial stress syndrome | 5 (4%) | 3 (4%) | 2 (5%) | Shin |
| Hamstring strain | 5 (4%) | 5 (7%) | 0 (0%) | Thigh |
| Gluteal strain | 5 (4%) | 2 (3%) | 3 (7%) | Buttocks |
| Disc pain/referred low back pain | 5 (4%) | 5 (7 %) | 0 (0%) | Low back |
| Hamstring tendinopathy | 4 (3%) | 1 (1%) | 3 (7%) | Thigh |
| Hip flexor strain | 2 (2%) | 1 (1%) | 1 (2%) | Hip |
| Iliotibial band syndrome | 3 (3%) | 2 (3%) | 1 (2%) | Knee |
| Knee meniscus injury | 3 (3%) | 3 (4%) | 0 (0%) | Knee |
| Flexor hallucis longus tendinopathy | 2 (2%) | 2 (3%) | 0 (0%) | Foot |
| Patellar tendon pain | 1 (1%) | 0 (0%) | 1 (2%) | Knee |
| Foot extensor tendinopathy | 1 (1%) | 0 (0%) | 1 (2%) | Foot |
| Metatarsalgia | 2 (2%) | 2 (3%) | 0 (0%) | Foot |
| Morton's Neuroma | 1 (1%) | 0 (0%) | 1 (2%) | Foot |
| Quadriceps Tendon Pain | 1 (1%) | 1 (1%) | 0 (0%) | Knee |
| Hip bursitis | 1 (1%) | 1 (1%) | 0 (0%) | Hip |
| Exertional lower leg compartment syndrome | 1 (1%) | 0 (0%) | 1 (2%) | Shin |
| Adductor magnus strain | 1 (1%) | 0 (0%) | 1 (2%) | Thigh |
| Peroneal tendon pain | 1 (1%) | 0 (0%) | 1 (2%) | Foot |
| Degenerative hip pain (OA) | 1 (1%) | 0 (0%) | 1 (2%) | Hip |
