# Supplementary material for: Running towards injury? A prospective investigation of factors associated with running injuries
Source: PLoS One. 2023 Aug 17;18(8):e0288814. doi: 10.1371/journal.pone.0288814 (PMC10434952; doi:10.1371/journal.pone.0288814)
Supplement: S3 Table — (DOCX) [file pone.0288814.s003.docx]

S3 Table. Results of the correlation analysis.

| Variable 1 | Variable 2 | Spearman's Rho |
| --- | --- | --- |
| Peak hip adduction | Hip adduction at initial contact* | 0.73 |
| Hip flexion at initial contact* | Peak hip flexion | 1.00 |
| Hip flexion at initial contact* | Pelvis anterior tilt at initial contact | 0.82 |
| Hip flexion at initial contact* | Pelvis anterior tilt at toe-off | 0.74 |
| Hip flexion at initial contact* | Peak pelvis anterior tilt | 0.75 |
| Hip flexion at initial contact* | Minimum anterior pelvis tilt | 0.74 |
| Hip flexion at toe-off* | Minimum hip flexion | 0.99 |
| Hip flexion at toe-off* | Pelvis anterior tilt at initial contact | 0.71 |
| Hip flexion at toe-off* | Pelvis anterior tilt at toe-off | 0.78 |
| Hip flexion at toe-off* | Peak pelvis anterior tilt | 0.76 |
| Hip flexion at toe-off* | Minimum anterior pelvis tilt | 0.70 |
| Peak hip flexion | Pelvis anterior tilt at initial contact | 0.82 |
| Peak hip flexion | Pelvis anterior tilt at toe-off | 0.76 |
| Peak hip flexion | Peak pelvis anterior tilt | 0.77 |
| Peak hip flexion | Minimum anterior pelvis tilt | 0.75 |
| Minimum hip flexion | Pelvis anterior tilt at initial contact | 0.72 |
| Minimum hip flexion | Pelvis anterior tilt at toe-off | 0.79 |
| Minimum hip flexion | Peak pelvis anterior tilt | 0.77 |
| Minimum hip flexion | Minimum anterior pelvis tilt | 0.72 |
| Hip internal rotation at initial contact | Peak hip internal rotation | 0.90 |
| Peak hip internal rotation | Minimum hip internal rotation | 0.78 |
| Knee varus at initial contact* | Knee varus at toe-off | 0.89 |
| Knee varus at initial contact* | Peak knee varus | 0.95 |
| Knee varus at initial contact* | Minimum knee varus | 0.85 |
| Knee varus at toe-off | Peak knee varus | 0.91 |
| Peak knee varus | Minimum knee varus | 0.86 |
| Minimum knee varus | Knee varus at toe-off | 0.82 |
| Peak pelvis contralateral rotation | Pelvis contralateral rotation at toe-off* | 0.97 |
| Peak thorax rotation to the contralateral side | Thorax rotation to contralateral side at toe-off* | 1.00 |
| Thorax rotation to contralateral side at initial contact | Minimum thorax rotation to the contralateral side* | 1.00 |

All variables were significantly correlated at the p < .01 level. * indicates that the variable was included in the multivariable analysis.
