# Supplementary material for: Running towards injury? A prospective investigation of factors associated with running injuries
Source: PLoS One. 2023 Aug 17;18(8):e0288814. doi: 10.1371/journal.pone.0288814 (PMC10434952; doi:10.1371/journal.pone.0288814)
Supplement: S4 Table — (DOCX) [file pone.0288814.s004.docx]

S4 Table. Results of the univariable analysis.

| **Variable** | Uninjured | Injured | Unadjusted | | | | Adjusted* | | | |
| --- | --- | --- | --- | --- | --- | --- | --- | --- | --- | --- |
|  | Mean ± SD | Mean ± SD | Sig. | OR | 95% C.I.for OR | | Sig. | OR | 95% C.I. for OR | |
| **Demographics** |  |  |  |  | Lower | Upper |  |  | Lower | Upper |
| Age (years) | 43.6 ± 9.3 | 43.4 ± 8.4 | 0.888 | 1.00 | 0.97 | 1.03 |  |  |  |  |
| BMI (kg/m2) | 24.2 ± 2.9 | 23.7 ± 2.9 | 0.188 | 0.94 | 0.86 | 1.03 | 0.094 | 0.92 | 0.83 | 1.02 |
| Female sex (reference is male) | 41 females (38%) | 43 females (37%) | 0.851 | 0.95 | 0.55 | 1.63 |  |  |  |  |
| **Training and injury history** | |  |  |  |  |  |  |  |  |  |
| History of RRI < 1 year ago | 38 (35%) | 57 (49%) | 0.041 | 1.75 | 1.02 | 2.99 | 0.05 | 1.72 | 1.00 | 2.95 |
| Average weekly mileage (km) | 36.4 ± 21.2 | 34.5 ± 19.4 | 0.481 | 1.00 | 0.98 | 1.01 |  |  |  |  |
| Self-reported average running pace (km/hr) | 11.3 ± 1.9 | 11.5 ± 1.6 | 0.289 | 1.09 | 0.93 | 1.27 | 0.222 | 1.11 | 0.94 | 1.32 |
| Running experience > 10 years  (Reference is <10 years’ experience) | 27 (25%) | 24 (21%) | 0.422 | 0.77 | 0.41 | 1.45 | 0.383 | 0.76 | 0.40 | 1.42 |
| **Clinical Tests** |  |  |  |  |  |  |  |  |  |  |
| Navicular drop (mm) | 9.0 ± 3.3 | 7.9 ± 2.9 | 0.005 | 0.88 | 0.81 | 0.96 | 0.004 | 0.26 | 0.11 | 0.65 |
| Navicular Drop > 10 mm (reference is navicular drop <10 mm) | 40 (37%) | 30 (26%) | 0.066 | 0.59 | 0.33 | 1.04 | 0.05 | 0.56 | 0.31 | 1.00 |
| Foot Posture Index: Pronated foot (reference is neutral foot) | 60 (56%) | 64 (55%) | 0.894 | 1.04 | 0.59 | 1.84 | 0.921 | 0.97 | 0.55 | 1.72 |
| Foot Posture Index: Supinated foot (reference is neutral foot) | 10 (9%) | 14 (12%) | 0.511 | 1.36 | 0.54 | 3.45 | 0.522 | 1.36 | 0.53 | 3.46 |
| Hip abduction strength (Nm/kg) | 1.70 ± 0.32 | 1.64 ± 0.30 | 0.17 | 0.55 | 0.24 | 1.29 | 0.149 | 0.52 | 0.21 | 1.27 |
| Hip extension strength (Nm/kg) | 1.91 ± 0.45 | 1.90 ± 0.45 | 0.904 | 0.97 | 0.54 | 1.73 | 0.923 | 0.97 | 0.53 | 1.78 |
| Plantar flexion strength (Nm/kg) | 0.58 ± 0.21 | 0.56 ± 0.21 | 0.36 | 0.56 | 0.16 | 1.96 | 0.353 | 0.54 | 0.15 | 1.97 |
| Knee extension strength (Nm/kg) | 1.33 ± 0.41 | 1.27 ± 0.37 | 0.289 | 0.69 | 0.35 | 1.37 | 0.253 | 0.65 | 0.30 | 1.37 |
| Knee flexion strength (Nm/kg) | 0.99 ± 0.28 | 0.93 ± 0.25 | 0.104 | 0.44 | 0.16 | 1.18 | 0.076 | 0.37 | 0.12 | 1.11 |
| Hip internal rotation ROM (°) | 39.4 ± 6.6 | 40.3 ± 6.6 | 0.305 | 1.02 | 0.98 | 1.06 | 0.278 | 1.02 | 0.98 | 1.07 |
| Hip external rotation ROM (°) | 37.1 ± 6.6 | 36.8 ± 5.7 | 0.749 | 0.99 | 0.95 | 1.04 | 0.721 | 0.99 | 0.95 | 1.04 |
| Hip extension ROM (°) | 12.1 ± 7.0 | 11.8 ± 7.2 | 0.766 | 0.99 | 0.96 | 1.03 | 0.824 | 1.00 | 0.96 | 1.03 |
| Ankle dorsiflexion ROM (°) | 40.2 ± 4.0 | 39.8 ± 4.2 | 0.423 | 0.97 | 0.91 | 1.04 | 0.415 | 0.97 | 0.91 | 1.04 |
| **Running kinetics** | |  |  |  |  |  |  |  |  |  |
| Peak sacrum accelerations (g) | -5.87 ± 1.66 | -5.80 ± 1.86 | 0.972 | 1.00 | 0.86 | 1.16 | 0.919 | 0.99 | 0.85 | 1.16 |
| Peak shank accelerations (g) | -7.26 ± 2.35 | -7.21 ± 2.09 | 0.389 | 1.05 | 0.94 | 1.18 | 0.472 | 1.05 | 0.93 | 1.18 |
| Sacrum rate of accelerations (g/s) | 305 ± 144 | 311 ± 172 | 0.808 | 1.00 | 1.00 | 1.00 | 0.706 | 1.00 | 1.00 | 1.00 |
| Shank rate of accelerations (g/s) | 600 ± 371 | 572 ± 317 | 0.544 | 1.00 | 1.00 | 1.00 | 0.68 | 1.00 | 1.00 | 1.00 |
| **Running kinematics** | |  |  |  |  |  |  |  |  |  |
| Ankle eversion at initial contact (°) | 2.0 ± 2.2 | 1.7 ± 2.1 | 0.411 | 0.95 | 0.84 | 1.07 | 0.405 | 0.95 | 0.84 | 1.08 |
| Ankle eversion at toe-off (°) | 0.1 ± 2.1 | -0.1 ± 2.0 | 0.445 | 0.95 | 0.83 | 1.08 | 0.408 | 0.95 | 0.83 | 1.08 |
| Ankle eversion inversion excursion (°) | 6.8 ± 2.1 | 6.5 ± 1.9 | 0.358 | 0.94 | 0.82 | 1.07 | 0.351 | 0.94 | 0.82 | 1.07 |
| Peak ankle eversion (°) | 6.7 ± 2.8 | 6.3 ± 2.5 | 0.198 | 0.94 | 0.85 | 1.04 | 0.184 | 0.93 | 0.84 | 1.03 |
| Minimum ankle eversion (°) | -0.1 ± 2.7 | -0.3 ± 1.9 | 0.421 | 0.95 | 0.83 | 1.08 | 0.39 | 0.94 | 0.82 | 1.08 |
| Ankle dorsiflexion at initial contact (°) | 10.4 ± 6.3 | 10.2 ± 6.0 | 0.806 | 1.00 | 0.95 | 1.04 | 0.727 | 0.99 | 0.95 | 1.04 |
| Ankle dorsiflexion at toe-off (°) | -14.5 ± 6.1 | -15. 2 ± 5.9 | 0.42 | 0.98 | 0.94 | 1.03 | 0.354 | 0.98 | 0.93 | 1.03 |
| Ankle dorsiflexion-plantarflexion excursion (°) | 41.9 ± 5.8 | 42.5 ± 5.6 | 0.426 | 1.02 | 0.97 | 1.07 | 0.374 | 1.02 | 0.97 | 1.07 |
| Peak ankle dorsiflexion (°) | 27.4 ± 4.0 | 27.3 ± 4.0 | 0.919 | 1.00 | 0.93 | 1.07 | 0.876 | 1.00 | 0.93 | 1.06 |
| Minimum ankle dorsiflexion (°) | -14.5 ± 6.1 | -15.2 ± 5.9 | 0.409 | 0.98 | 0.94 | 1.03 | 0.342 | 0.98 | 0.93 | 1.02 |
| Ankle internal rotation at initial contact (°) | -7.4 ± 8.4 | -6.6 ± 8.5 | 0.487 | 1.01 | 0.98 | 1.04 | 0.488 | 1.01 | 0.98 | 1.04 |
| Ankle internal rotation at toe-off (°) | -0.3 ± 8.2 | 0.6 ± 8.2 | 0.445 | 1.01 | 0.98 | 1.05 | 0.403 | 1.01 | 0.98 | 1.05 |
| Ankle internal rotation external rotation excursion (°) | 25.1 ± 5.5 | 25.0 ± 5.3 | 0.358 | 0.94 | 0.82 | 1.07 | 0.984 | 1.00 | 0.95 | 1.05 |
| Peak ankle internal rotation (°) | 0.5 ± 8.0 | 1.3 ± 7.8 | 0.429 | 1.01 | 0.98 | 1.05 | 0.392 | 1.02 | 0.98 | 1.05 |
| Minimum ankle internal rotation (°) | -24.6 ± 7.9 | -23.7 ± 7.8 | 0.401 | 1.02 | 0.98 | 1.05 | 0.39 | 1.02 | 0.98 | 1.05 |
| Foot strike pattern: MFS (reference is RFS) | 10 (9%) | 14 (12%) | 0.535 | 1.31 | 0.56 | 3.11 | 0.484 | 1.37 | 0.57 | 3.26 |
| Foot strike pattern: FFS (reference is RFS) | 7 (6%) | 6 (5%) | 0.705 | 0.80 | 0.26 | 2.48 | 0.726 | 0.81 | 0.26 | 2.58 |
| Hip adduction at initial contact (°) | 10.1 ± 4.0 | 9.1 ± 3.8 | 0.054 | 0.93 | 0.87 | 1.00 | 0.062 | 0.93 | 0.86 | 1.00 |
| Hip adduction at toe-off (°) | 1.1 ± 3.4 | -0.0 ± 3.7 | 0.022 | 0.92 | 0.85 | 0.99 | 0.024 | 0.92 | 0.85 | 0.99 |
| Hip adduction abduction excursion (°) | 13.8 ± 4.3 | 13.8 ± 3.9 | 0.977 | 1.00 | 0.94 | 1.07 | 0.934 | 1.00 | 0.94 | 1.07 |
| Peak hip adduction (°) | 14.7 ± 4.6 | 13.6 ± 4.0 | 0.057 | 0.94 | 0.88 | 1.00 | 0.067 | 0.94 | 0.88 | 1.00 |
| Minimum hip adduction (°) | 0.9 ± 3.4 | -0.18 ± 3.7 | 0.057 | 0.94 | 0.88 | 1.00 | 0.023 | 0.91 | 0.85 | 0.99 |
| Hip flexion at initial contact (°) | 38.9 ± 6.5 | 37.8 ± 6.6 | 0.189 | 0.97 | 0.94 | 1.01 | 0.166 | 0.97 | 0.93 | 1.01 |
| Hip flexion at toe-off (°) | -5.0 ± 6.1 | -6.2 ± 6.0 | 0.113 | 0.97 | 0.92 | 1.01 | 0.071 | 0.96 | 0.91 | 1.00 |
| Hip flexion extension excursion (°) | 44.2 ± 5.5 | 44.5 ± 5.6 | 0.821 | 1.01 | 0.96 | 1.05 | 0.764 | 1.01 | 0.96 | 1.06 |
| Peak hip flexion (°) | 39.1 ± 6.5 | 38.0 ± 6.6 | 0.239 | 0.98 | 0.94 | 1.02 | 0.207 | 0.97 | 0.93 | 1.02 |
| Minimum hip flexion (°) | -5.2 ± 6.0 | -6.4 ± 5.9 | 0.133 | 0.97 | 0.92 | 1.01 | 0.085 | 0.96 | 0.91 | 1.01 |
| Hip internal rotation at initial contact (°) | -1.5 ± 6.3 | -0.1 ± 6.4 | 0.107 | 1.04 | 0.99 | 1.08 | 0.054 | 1.05 | 1.00 | 1.10 |
| Hip internal rotation at toe-off (°) | -8.6 ± 6.4 | -8.2 ± 6.7 | 0.573 | 1.01 | 0.97 | 1.05 | 0.475 | 1.02 | 0.97 | 1.06 |
| Hip internal rotation external rotation excursion (°) | 10.8 ± 3.8 | 11.7 ± 3.8 | 0.066 | 1.07 | 1.00 | 1.15 | 0.052 | 1.08 | 1.00 | 1.16 |
| Peak hip internal rotation (°) | 0.9 ± 6.2 | 2.0 ± 6.1 | 0.172 | 1.03 | 0.99 | 1.08 | 0.105 | 1.04 | 0.99 | 1.09 |
| Minimum hip internal rotation (°) | -9.8 ± 5.9 | -9.7 ± 6.1 | 0.817 | 1.01 | 0.96 | 1.05 | 0.693 | 1.01 | 0.97 | 1.06 |
| Knee varus at initial contact (°) | -2.9 ± 2.6 | -1.2 ± 3.11 | 0.001 | 1.19 | 1.08 | 1.32 | 0.001 | 1.20 | 1.08 | 1.33 |
| Knee varus at toe-off (°) | -3.9 ± 3.0 | -2.7 ± 3.1 | 0.004 | 1.15 | 1.05 | 1.26 | 0.004 | 1.15 | 1.04 | 1.26 |
| Knee varus valgus excursion (°) | 3.9 ± 1.6 | 4.3 ± 1.7 | 0.06 | 1.17 | 0.99 | 1.38 | 0.051 | 1.18 | 1.00 | 1.40 |
| Peak knee varus (°) | -2.1 ± 2.7 | -0.8 ± 3.1 | 0.003 | 1.16 | 1.05 | 1.28 | 0.003 | 1.16 | 1.05 | 1.28 |
| Minimum knee varus (°) | -5.9 ± 2.9 | -5.00 ± 3.5 | 0.032 | 1.10 | 1.01 | 1.19 | 0.038 | 1.10 | 1.01 | 1.19 |
| Knee flexion at initial contact (°) | 17.8 ± 5.1 | 17.6 ± 4.7 | 0.757 | 0.99 | 0.94 | 1.05 | 0.886 | 1.00 | 0.94 | 1.05 |
| Knee flexion at toe-off (°) | 16.0 ± 6.0 | 15.5 ± 6.5 | 0.553 | 0.99 | 0.95 | 1.03 | 0.534 | 0.99 | 0.94 | 1.03 |
| Knee flexion extension excursion (°) | 30.9 ± 5.4 | 31.0 ± 5.5 | 0.627 | 1.01 | 0.97 | 1.06 | 0.635 | 1.01 | 0.96 | 1.06 |
| Peak knee flexion (°) | 44.2 ± 4.4 | 44.3 ± 4.7 | 0.868 | 1.01 | 0.95 | 1.07 | 0.845 | 1.01 | 0.95 | 1.07 |
| Minimum knee flexion (°) | 13.5 ± 4.4 | 13.2 ± 5.1 | 0.698 | 0.99 | 0.94 | 1.04 | 0.733 | 0.99 | 0.94 | 1.05 |
| Knee internal rotation at initial contact (°) | 4.3 ± 6.4 | 4.4 ± 6.4 | 0.853 | 1.00 | 0.96 | 1.05 | 0.897 | 1.00 | 0.96 | 1.05 |
| Knee internal rotation at toe-off (°) | 3.8 ± 6.6 | 3.7 ± 6.3 | 0.86 | 1.00 | 0.96 | 1.04 | 0.752 | 0.99 | 0.95 | 1.04 |
| Knee internal rotation external rotation excursion (°) | 20.9 ± 4.5 | 22.4 ± 5.4 | 0.024 | 1.07 | 1.01 | 1.13 | 0.024 | 1.07 | 1.01 | 1.13 |
| Peak knee internal rotation (°) | 22.9 ± 6.7 | 24.1 ± 7.7 | 0.228 | 1.02 | 0.99 | 1.06 | 0.269 | 1.02 | 0.98 | 1.06 |
| Minimum knee internal rotation (°) | 2.0 ± 6.3 | 1.7 ± 5.8 | 0.678 | 0.99 | 0.95 | 1.04 | 0.602 | 0.99 | 0.95 | 1.03 |
| Pelvis contralateral drop at initial contact (°) | 2.2 ± 2.7 | 1.9 ± 2.3 | 0.371 | 0.95 | 0.86 | 1.06 | 0.393 | 0.96 | 0.86 | 1.06 |
| Pelvis contralateral drop at toe-off (°) | -4.8 ± 2.7 | -4.9 ± 2.7 | 0.733 | 0.98 | 0.89 | 1.09 | 0.725 | 0.98 | 0.89 | 1.09 |
| Pelvis contralateral-ipsilateral drop excursion (°) | 9.8 ± 3.4 | 9.4 ± 2.77 | 0.307 | 0.96 | 0.88 | 1.04 | 0.276 | 0.95 | 0.87 | 1.04 |
| Peak pelvis contralateral drop (°) | 4.9 ± 2.7 | 4.4 ± 2.5 | 0.203 | 0.94 | 0.85 | 1.04 | 0.2 | 0.94 | 0.85 | 1.04 |
| Minimum pelvis contralateral drop (°) | -5.0 ± 2.6 | -5.0 ± 2.6 | 0.932 | 1.00 | 0.90 | 1.10 | 0.942 | 1.00 | 0.90 | 1.11 |
| Pelvis anterior tilt at initial contact (°) | 15.6 ± 5.7 | 14.6 ± 5.2 | 0.168 | 0.97 | 0.92 | 1.02 | 0.133 | 0.96 | 0.92 | 1.01 |
| Pelvis anterior tilt at toe-off (°) | 18.1 ± 5.7 | 17.0 ± 4.9 | 0.14 | 0.96 | 0.92 | 1.01 | 0.103 | 0.96 | 0.91 | 1.01 |
| Pelvis anterior-posterior tilt excursion (°) | 7.6 ± 2.1 | 7.5 ± 2.0 | 0.626 | 0.97 | 0.85 | 1.10 | 0.586 | 0.96 | 0.85 | 1.10 |
| Peak pelvis anterior tilt (°) | 18.4 ± 5.7 | 17.4 ± 5.0 | 0.159 | 0.97 | 0.92 | 1.01 | 0.12 | 0.96 | 0.91 | 1.01 |
| Minimum pelvis anterior tilt (°) | 10.8 ± 5.9 | 9.9 ± 5.1 | 0.056 | 0.94 | 0.87 | 1.00 | 0.187 | 0.97 | 0.92 | 1.02 |
| Pelvis contralateral rotation at initial contact (°) | -2.8 ± 3.6 | -3.5 ± 4.6 | 0.169 | 0.96 | 0.90 | 1.02 | 0.117 | 0.95 | 0.89 | 1.01 |
| Pelvis contralateral rotation at toe-off (°) | 3.6 ± 4.1 | 2.3 ± 3.9 | 0.018 | 0.92 | 0.86 | 0.99 | 0.014 | 0.92 | 0.85 | 0.98 |
| Pelvis contralateral rotation external rotation excursion (°) | 9.2 ± 3.4 | 9.1 ± 3.7 | 0.85 | 0.99 | 0.92 | 1.07 | 0.88 | 0.99 | 0.91 | 1.08 |
| Peak pelvis contralateral rotation (°) | 3.8 ± 3.7 | 2.8 ± 3.7 | 0.029 | 0.92 | 0.86 | 0.99 | 0.024 | 0.92 | 0.85 | 0.99 |
| Minimum pelvis contralateral rotation (°) | -5.3 ± 3.6 | -6.4 ± 4.2 | 0.056 | 0.94 | 0.87 | 1.00 | 0.042 | 0.93 | 0.87 | 1.00 |
| Thorax contralateral side flexion at initial contact (°) | -2.8 ± 2.4 | -3.2 ± 2.4 | 0.314 | 0.95 | 0.85 | 1.06 | 0.332 | 0.95 | 0.85 | 1.06 |
| Thorax contralateral side flexion at toe-off (°) | 0.6 ± 2.3 | 0.8 ± 2.4 | 0.612 | 1.03 | 0.92 | 1.15 | 0.619 | 1.03 | 0.92 | 1.15 |
| Thorax contralateral-ipsilateral side flexion excursion (°) | 5.1 ± 1.7 | 5.6 ± 2.2 | 0.077 | 1.13 | 0.99 | 1.29 | 0.084 | 1.13 | 0.98 | 1.29 |
| Peak thorax contralateral side flexion (°) | 0.7 ± 2.3 | 0.9 ± 2.3 | 0.566 | 1.03 | 0.92 | 1.16 | 0.572 | 1.03 | 0.92 | 1.16 |
| Minimum thorax contralateral side flexion (°) | -4.4 ± 2.4 | -4.7 ± 2.2 | 0.311 | 0.94 | 0.84 | 1.06 | 0.325 | 0.94 | 0.84 | 1.06 |
| Thorax forward flexion at initial contact (°) | 7.4 ± 4.7 | 8.1 ± 4.6 | 0.288 | 1.03 | 0.97 | 1.09 | 0.292 | 1.03 | 0.97 | 1.09 |
| Thorax forward flexion at toe-off (°) | 7.1 ± 4.8 | 7.5 ± 4.8 | 0.615 | 1.01 | 0.96 | 1.07 | 0.292 | 1.03 | 0.97 | 1.09 |
| Thorax flexion-extension excursion (°) | 3.7 ± 1.3 | 3.9 ± 1.4 | 0.268 | 1.12 | 0.92 | 1.36 | 0.204 | 1.15 | 0.93 | 1.42 |
| Peak thorax forward flexion (°) | 10.2 ± 4.6 | 10.8 ± 4.7 | 0.346 | 1.03 | 0.97 | 1.09 | 0.344 | 1.03 | 0.97 | 1.09 |
| Minimum thorax forward flexion (°) | 6.5 ± 4.7 | 6.9 ± 4.6 | 0.449 | 1.02 | 0.97 | 1.08 | 0.458 | 1.02 | 0.97 | 1.08 |
| Thorax rotation towards contralateral side at initial contact (°) | -13.0 ± 4.4 | -13.8 ± 4.1 | 0.222 | 0.96 | 0.91 | 1.02 | 0.165 | 0.96 | 0.90 | 1.02 |
| Thorax rotation towards contralateral side at toe-off (°) | 14.6 ± 4.7 | 13.7 ± 4.1 | 0.147 | 0.96 | 0.90 | 1.02 | 0.14 | 0.95 | 0.89 | 1.02 |
| Thorax rotation contralateral-ipsilateral rotation excursion (°) | 27.7 ± 6.3 | 27.5 ± 6.3 | 0.868 | 1.00 | 0.96 | 1.04 | 0.937 | 1.00 | 0.95 | 1.05 |
| Peak thorax rotation towards contralateral side (°) | 14.6 ± 4.7 | 13.7 ± 4.1 | 0.141 | 0.96 | 0.90 | 1.02 | 0.134 | 0.95 | 0.89 | 1.02 |
| Minimum thorax rotation towards contralateral side (°) | -13.1 ± 4.4 | -13.8 ± 4.6 | 0.22 | 0.96 | 0.91 | 1.02 | 0.163 | 0.96 | 0.90 | 1.02 |
| **Spatiotemporal parameters** | |  |  |  |  |  |  |  |  |  |
| Stride length (metres) | 2.18 ± 0.32 | 2.15 ± 0.28 | 0.402 | 0.69 | 0.29 | 1.65 | 0.39 | 0.68 | 0.28 | 1.64 |
| Flight time (milliseconds) | 441 ± 38 | 434 ± 39 | 0.209 | 1.00 | 0.99 | 1.00 | 0.209 | 1.00 | 0.99 | 1.00 |
| Stance time (milliseconds) | 269 ± 38 | 265 ± 28 | 0.405 | 1.00 | 0.99 | 1.01 | 0.314 | 1.00 | 0.99 | 1.00 |
| Stride rate (strides/min) | 84.86 ± 4.80 | 85.63 ± 4.17 | 0.207 | 1.04 | 0.98 | 1.10 | 0.215 | 1.04 | 0.98 | 1.10 |
| Step time (milliseconds) | 708 ± 43 | 707 ± 39 | 0.773 | 1.00 | 0.99 | 1.01 | 0.609 | 1.00 | 0.99 | 1.01 |

Abbreviations: SD = standard deviation, Sig = significant, CI = confidence interval, OR = odds ratio, ROM = range of motion. * Adjusted for age, sex and weekly mileage. The following denote the direction of the moment: Ankle dorsiflexion (positive), plantar flexion (negative), ankle inversion (positive), ankle eversion (negative), ankle internal rotation (positive), ankle external rotation (negative), knee flexion(positive), knee extension (negative), knee varus (positive), knee valgus (negative), knee internal rotation (positive), knee external rotation (negative), hip flexion (positive), hip extension (negative), hip adduction (positive), hip abduction (negative), hip internal rotation (positive), hip external rotation (negative), thorax anterior tilt (positive), thorax posterior tilt (negative), thorax drop to contralateral side (positive), thorax drop to ipsilateral; side (negative), thorax rotation to contralateral side (positive), thorax rotation to ipsilateral side (negative), pelvis anterior tilt (positive), pelvis posterior tilt (negative), pelvis drop to contralateral side (positive), pelvis drop to ipsilateral; side (negative), pelvis rotation to contralateral side (positive), pelvis rotation to ipsilateral side (negative)
